# A novel deep learning prognostic system improves survival predictions for stage III non‐small cell lung cancer

**DOI:** 10.1002/cam4.4782

**Published:** 2022-05-02

**Authors:** Linlin Yang, Xinyu Fan, Wenru Qin, Yiyue Xu, Bing Zou, Bingjie Fan, Shijiang Wang, Taotao Dong, Linlin Wang

**Affiliations:** ^1^ Cheeloo College of Medicine Shandong University Jinan China; ^2^ Department of Radiation Oncology Shandong Cancer Hospital and Institute, Shandong First Medical University and Shandong Academy of Medical Science Jinan China; ^3^ Weifang Medical University Weifang China; ^4^ Department of Obstetrics and Gynecology Qilu Hospital of Shandong University Jinan China

**Keywords:** deep learning, non‐small cell lung cancer, prognosis, survival analysis

## Abstract

**Background:**

Accurate prognostic prediction plays a crucial role in the clinical setting. However, the TNM staging system fails to provide satisfactory individual survival prediction for stage III non‐small cell lung cancer (NSCLC). The performance of the deep learning network for survival prediction in stage III NSCLC has not been explored.

**Objectives:**

This study aimed to develop a deep learning‐based prognostic system that could achieve better predictive performance than the existing staging system for stage III NSCLC.

**Methods:**

In this study, a deep survival learning model (DSLM) for stage III NSCLC was developed based on the Surveillance, Epidemiology, and End Results (SEER) database and was independently tested with another external cohort from our institute. DSLM was compared with the Cox proportional hazard (CPH) and random survival forest (RSF) models. A new prognostic system for stage III NSCLC was also proposed based on the established deep learning model.

**Results:**

The study included 16,613 patients with stage III NSCLC from the SEER database. DSLM showed the best performance in survival prediction, with a C‐index of 0.725 in the validation set, followed by RSF (0.688) and CPH (0.683). DSLM also showed C‐indices of 0.719 and 0.665 in the internal and real‐world external testing datasets, respectively. In addition, the new prognostic system based on DSLM (AUROC = 0.744) showed better performance than the TNM staging system (AUROC = 0.561).

**Conclusion:**

In this study, a new, integrated deep learning‐based prognostic model was developed and evaluated for stage III NSCLC. This novel approach may be valuable in improving patient stratification and potentially provide meaningful prognostic information that contributes to personalized therapy.

## Graphical abstract

A newly integrated deep learning based prognostic model was developed and evaluated for stage III NSCLC, and was shown to have better performance than the TNM staging system. This novel approach may be valuable in improving patient stratification and potentially provide meaningful prognostic information that contributes to personalised therapy.

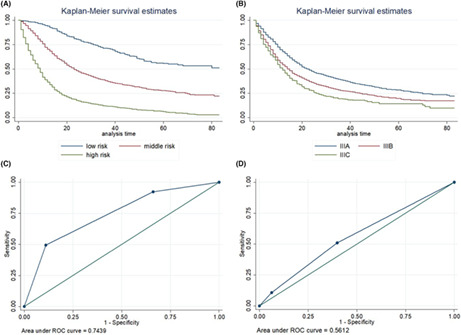

## 1 INTRODUCTION

Lung cancer is the second most commonly diagnosed cancer and the leading cause of cancer‐related death, with 80%–85% patients diagnosed with non‐small cell lung cancer (NSCLC).^1^, ^2^ Approximately 25%–40% of NSCLC cases are diagnosed at stage III disease, which demonstrates a more invasive clinical course and poorer prognosis than early‐stage disease, with two‐thirds of patients dying within 5 years.^3^, ^4^ Precise survival prediction plays a crucial role in improving risk stratification and patient survival. However, it is challenging to provide accurate prognostic information based on the currently used TNM staging system (8th edition of the tumor, node, and metastasis classification).^5^ Stage III NSCLC is characterized by heterogeneous features but is only canonically classified into IIIA, IIIB, and IIIC according to the TNM staging system or simply classified into resectable and unresectable diseases in clinical practice. In addition, the wide disparities in overall survival within the same stage suggest that other factors are important, including age, smoking, histology, differentiation, and treatment choices.^6^, ^7^, ^8^ Thus, a new comprehensive stratification system based on multiple prognostic factors is necessary for stage III NSCLC.

Various methods have been proposed to build a prognostic model by integrating multiple factors. Traditionally, most prognostic models have been based on regression models. For example, the Cox proportional hazard model (CPH) has been widely employed for such prognostic tasks, among which the nomogram is used to quantify risk and derive the risk probability of a specific event by combining significant clinical characteristics.^9^, ^10^, ^11^ Yet, these models can only detect the linear relationships between features and outcomes through fitting linear models. Consequently, a machine learning method with less restrictive model assumptions, namely, the random survival forest (RSF), has become attractive as a predictive tool.^12^, ^13^ However, accurate prediction via RSF heavily relies on the selection and parametrization of features based on prior biological knowledge. Moreover, RSF is considered to have a high risk of overfitting when analyzing large datasets and is unable to extrapolate the data.^14^

Deep learning‐based methods can handle large, complex, and heterogeneous data, thus allowing the solution of problems that cannot be properly addressed by traditional approaches.^15^, ^16^, ^17^ These methods have been applied successfully in biomedicine, including medical image segmentation and reading,^18^, ^19^ pathological diagnoses,^20^, ^21^ and cancer genomics.^22^, ^23^ In addition, the utility of these frameworks for survival prediction also has been examined. For example, Lee et al.^24^ demonstrated that DeepHIT, a deep neural network that directly learns the time‐to‐event distributions without making assumptions regarding the underlying stochastic process, could serve as a useful tool for survival prediction through a set of experiments conducted on a cohort of 5883 patients with adult cystic fibrosis. In another study, Kim et al.^25^ developed a deep learning network model for oral squamous cell carcinoma based on DeepSurv and achieved high C‐indices of 0.810 and 0.781 for the training and testing sets, respectively. However, no large‐sample research has focused on prognostication in patients with stage III NSCLC.

In this study, a deep survival learning model (DSLM) for stage III NSCLC survival prediction was developed using data from the Surveillance, Epidemiology, and End Results (SEER) dataset, and showed better performance compared to CPH and RSF. Moreover, an external test was performed on an independent cohort of 172 patients from Shandong Cancer Hospital and Institute. Lastly, we proposed a new prognostic system based on the DSLM, which achieved significant improvement in stage III NSCLC prognostication.

## 2 MATERIALS AND METHODS

### 2.1 Data source and study population

We selected patients from the SEER database which collects cancer incidence and survival data from population‐based cancer registries covering approximately 48% of the United States. A query of the SEER database was performed for primary lung and bronchus cancer using the “International Classification of Disease for Oncology, Third Edition (ICD‐O‐3).” This study enrolled patients with pathologically confirmed primary stage III NSCLC diagnosed between 2012 and 2015. Cases of multiple tumors, unknown tumor size, and incomplete follow‐up information were excluded. A real‐world dataset of newly diagnosed stage III NSCLC, managed at the Shandong Cancer Hospital and Institute between January 2012 and December 2016, was also retrospectively collected following approval by the institutional review board of the Shandong Cancer Hospital and Institute.

### 2.2 Clinical information and data processing

Among eligible cases, patient demographics, baseline information, tumor characteristics, first course of treatment, and follow‐up information were collected. The TNM stages of all eligible patients were redefined according to the 8th edition of the TNM staging to reflect current guideline recommendations. We removed strongly correlated variables using correlation matrix analyses. Overall survival, defined as the time between diagnosis and loss to follow‐up or death from any cause (loss to follow‐up and death were assigned values of 0 and 1, respectively), was used as the response variable for the survival analyses.

### 2.3 Deep survival neural network

The DSLM in this study was developed based on the neural multitask logistic regression model (N‐MTLR).^26^ This model was used to transform patient follow‐up time into a series of time points with the time point corresponding to the event (death) was assigned a value of 1. First, DSLM was applied to the stage III NSCLC dataset to make the prediction. Thereafter, patient samples were classified into three subgroups with different prognostic statuses, namely high‐risk, middle‐risk, and low‐risk, according to the values of the risk scores.

### 2.4 Baseline survival models for comparison

The baseline models including CPH and RSF were compared to the deep learning model for survival prediction in the provided dataset. CPH is a semiparametric model for survival analysis which estimates the risk function of the event occurring for patients based on observed features using a linear function. RSF is a nonlinear machine learning model used to analyze right‐censored survival data by generating ensemble estimates for the cumulative hazard function based on a nonparametric tree‐ensemble method.^12^ In this study, the RSF model was constructed with a tree count of 200, depth of two variables, and minimum node size of 5. Furthermore, all potential risk factors were ranked by their importance via RSF (Table S1).

### 2.5 Evaluation of models and statistical analyses

Model performance was evaluated using the concordance index (C‐index). The integrated Brier score (IBS), another measure for assessing predictive performance, was calculated to assess the overall error values across multiple time points. Generally, a useful model has an IBS below 0.25, with lower values indicating better predictive performance. Receiver operating characteristics and the area under the curve (AUROC) were used to compare predictive accuracy. Log‐rank tests were used for the comparison of different curves. A *p* value <0.05 was considered statistically significant. The training, validation, and testing procedures for the deep learning models were conducted with Pytorch. The Python (R Foundation for Statistical Computing) scikit‐learn and pandas packages were used for data analysis and the Python matplotlib was used for plotting graphs. The STATA statistical software package (StataCorp, 2015, Stata: Release 14. Statistical Software) was used for other conventional statistical analyses.

## 3 RESULTS

### 3.1 Patient characteristics

**FIGURE 1:**
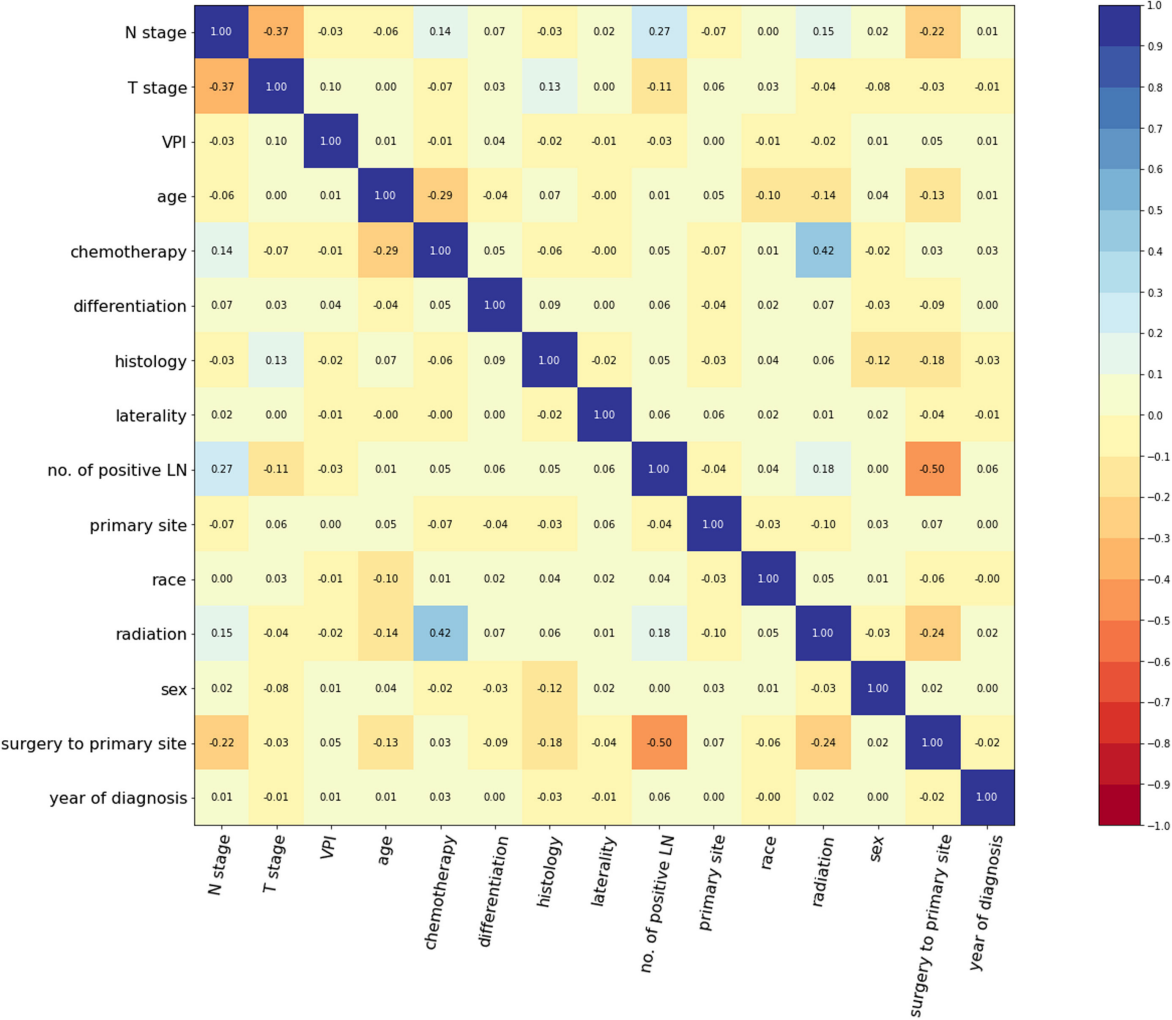
Correlation matrix of 15 selected variables. The matrix displays the correlation between variables. Each cell in the matrix shows the correlation coefficient between two variables. The main diagonal with several values of 1.00 going from the top left to the bottom right shows that each variable is perfectly correlated with itself. The matrix is symmetric, with the correlation above and below the main diagonal being mirror images of each other. LN, lymph nodes; VPI, visceral pleural invasion

According to the inclusion criteria, 16,613 patients with primary stage III NSCLC between 2012 and 2015 in the SEER database were enrolled in the study. Fifteen variables were selected for further analyses, including year of diagnosis, age, sex, race, T stage, N stage, primary site, laterality, histology, differentiation, number of positive lymph nodes, visceral pleural invasion, surgery to primary site, chemotherapy and radiotherapy. The correlation matrix was plotted for 15 selected variables (Figure 1). The median age was 68 (interquartile range [IQR] 61–76) years, and the majority of patients were white (13,169 [79.27%]). The median follow‐up time was 16 (IQR, 6–39) months. There were 13,027 (78.41%) patients with events (all‐cause death) during the follow‐up time. We used an independent dataset comprising 172 patients with stage III NSCLC diagnosed between January 2012 and December 2016 in Shandong Cancer Hospital and Institute. A total of 96 (55.81%) patients had events over a median follow‐up time of 33.5 (IQR 16–61.5) months. The patients' demographics and main baseline clinical characteristics are shown in Table 1.

**TABLE 1:** Patients' demographics and main baseline clinical characteristics

Characteristics	Patients, No. (%)	
	SEER (*N* = 16,613)	Shandong cancer hospital (*N* = 172)
Age at diagnosis, median (IQR), years	68 (61–76)	59 (52–65)
Race		
White	13,169 (79.27)	0
Black	2235 (13.45)	0
Asian or Pacific Islander	1109 (6.68)	172 (100)
American Indian/Alaska native	100 (0.60)	0
Sex		
Male	9361 (56.35)	133 (77.33)
Female	7252 (43.65)	39 (22.67)
Histology		
Adenocarcinoma	7925 (47.70)	84 (48.84)
Squamous cell carcinoma	7382 (44.44)	80 (46.51)
NSCLC, NOS	1306 (7.86)	8 (4.65)
Stage		
IIIA	8398 (50.55)	99 (57.56)
IIIB	6780 (40.81)	63 (36.63)
IIIC	1435 (8.64)	10 (5.81)
Laterality		
Left	6560 (39.49)	85 (49.42)
Right	10,053 (60.51)	87 (50.58)
Primary site		
Main bronchus	714 (4.30)	0
Upper lobe	9771 (58.82)	93 (54.07)
Middle lobe	729 (4.39)	13 (7.56)
Lower lobe	4447 (26.77)	66 (38.37)
Overlapping lesion	246 (1.48)	0
Lung, NOS	706 (4.25)	0
Differentiation		
Well differentiated	635 (3.82)	7 (4.07)
Moderately differentiated	3499 (21.06)	47 (27.33)
Poorly differentiated	5794 (34.88)	44 (25.58)
Undifferentiated	145 (0.87)	1 (0.58)
Unknown	6540 (39.37)	73 (42.44)
VPI		
Yes	1350 (8.13)	45 (26.16)
No	2323 (13.98)	66 (38.37)
Unknown	12,940 (77.89)	61 (35.47)
Surgery to primary site		
Yes	3791 (22.82)	63 (36.63)
No	12,822 (77.18)	109 (63.37)

Abbreviations: IQR, interquartile range; NOS, not otherwise specified; NSCLC, non‐small cell lung cancer; VPI, visceral pleural invasion.

### 3.2 Training and validation of the DSLM

The whole SEER dataset was split into training (*n* = 10,795, 65%), validation (*n* = 2907, 17.5%), and testing (*n* = 2907, 17.5%) sets, with five duplicates were dropped. The final deep learning network comprised four layers with 70 neurons in each layer. The ReLU activation was used for each layer. During training, we used the Adam optimizer to improve robustness. Grid searching was used to select optimal hyperparameters. Moreover, L2 regularization and dropout were used to prevent overfitting. Finally, DSLM achieved the best performance with a learning rate of 0.001, L2 regularization of 1e‐2, L2 smoothing of 1e‐2, and dropout rate of 0.2. The values of the loss function decreased from 44,000 to 32,988 after 2000 iterations (Figure S1).

**FIGURE 2:**
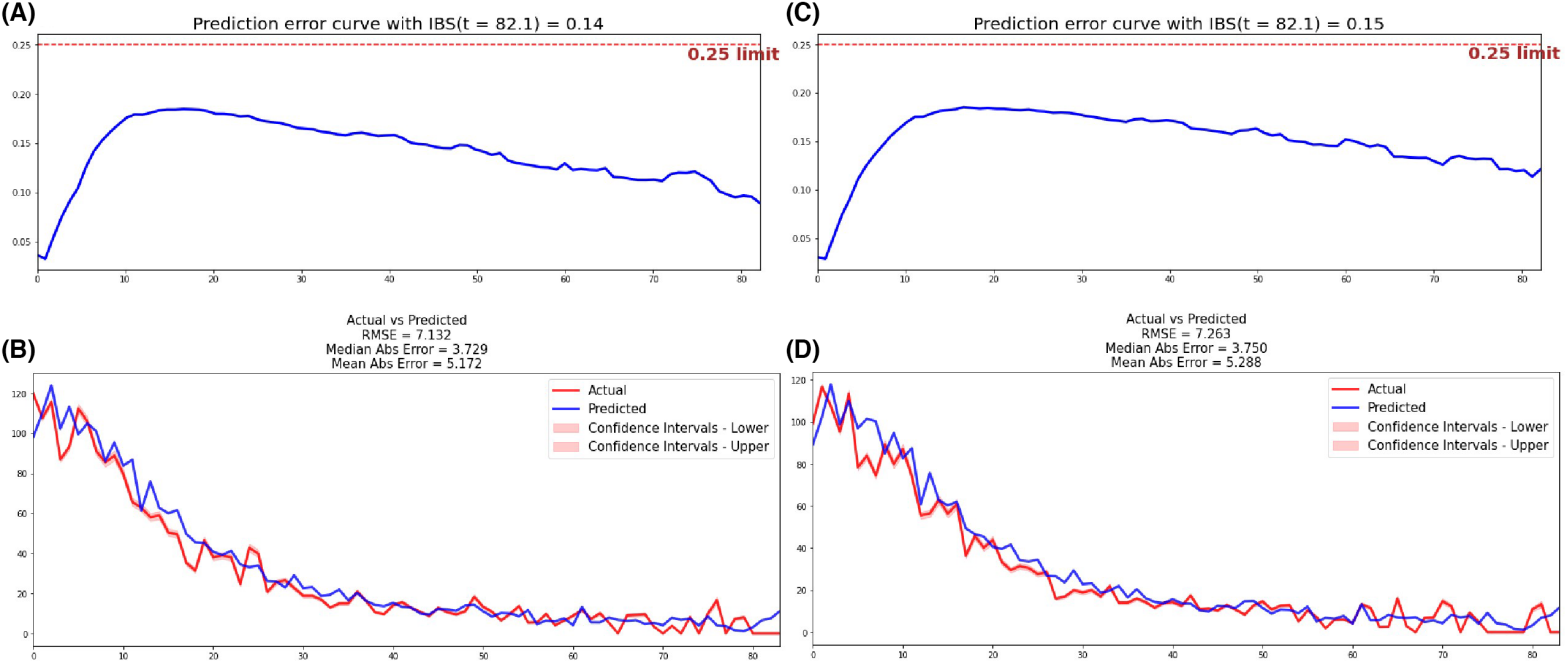
Prediction error curve with IBS and the calibration curves for DSLM. (A) DSLM had an IBS of 0.14 in the validation set. (B) Comparison of the actual and predicted number of patient deaths over the entire follow‐up. DSLM yielded a median absolute error of 3.729 and a mean absolute error of 5.172 in the validation set. (C) DSLM had an IBS of 0.15 in the testing set. (D) DSLM yielded a median absolute error of 3.750 and a mean absolute error of 5.288 in the testing set. The red line indicates the actual number of patient deaths, while the blue line indicated the predicted number of patient deaths. IBS, integrated Brier score; DSLM, deep survival learning model

DSLM showed the best performance in survival prediction with a C‐index of 0.725 and IBS of 0.14 in the validation set (Figure 2A), followed by RSF (C‐index = 0.688, IBS = 0.16, Figure S2A) and CPH (C‐index = 0.688, IBS = 0.17, Figure S2B). Additionally, the calibration curves of comparison between actual and predicted for DSLM yielded a median absolute error of 3.729 and a mean absolute error of 5.172 in the validation set (Figure 2B). To validate this model and address potential overfitting, DSLM was tested with an independent testing set. A C‐index of 0.719 and IBS of 0.15 (Figure 2C) were still achieved. Additionally, the calibration curves of DSLM showed better accuracy (the mean absolute error of 5.228) in the testing set (Figure 2D) than RSF (the mean absolute error of 7.092, Figure S3A) and CPH (the mean absolute error of 8.087, Figure S3B). Overall, these metrics confirmed the high performance and discriminatory ability of the deep learning‐based prognostic model.

### 3.3 Evaluation of the DSLM in an external testing set

To validate the general applicability of this deep learning‐based prognostic model, DSLM was tested in the external testing set comprising 172 stage III NSCLC patients from the Shandong Cancer Hospital and Institute. A C‐index of 0.665 was still achieved with the DSLM, which improved the performance of CPH (C‐index = 0.629) and RSF (C‐index = 0.635) in the external testing set.

### 3.4 New prognostic system based on DSLM

**FIGURE 3:**
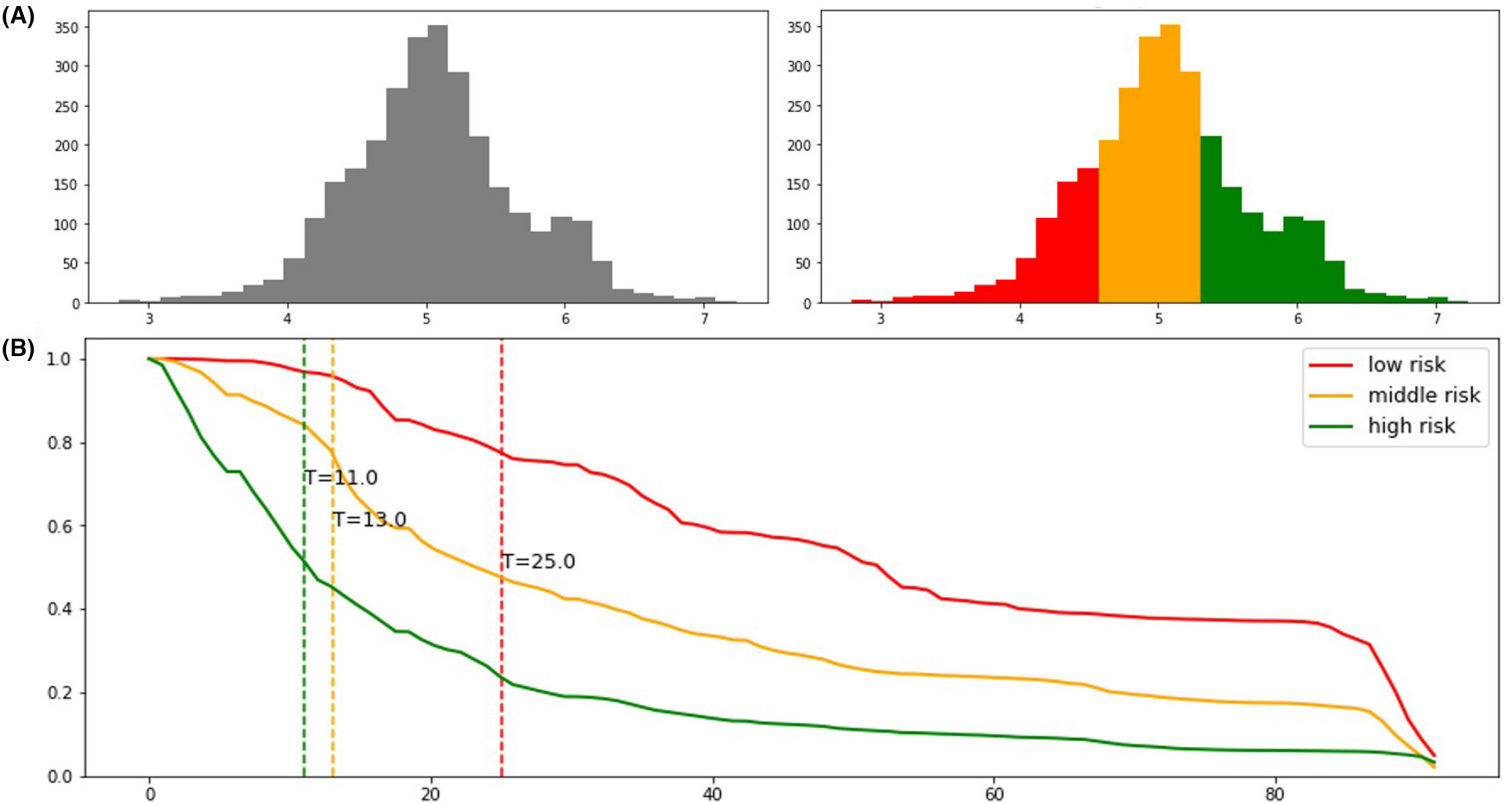
Three risk groups for patients in the testing set. (A) The left panel represents the overall risk score distribution of all patients enrolled in the testing set. The right panel represents the three different prognostic groups of patients based on their risk scores extracted by DSLM. (B) Personalized survival curve for patients in different risk groups. The survival curves for three patients from different risk groups were plotted and showed a significant difference in prognosis. Patients with an event were selected to visualize the actual time of the event. DSLM, deep survival learning model

**FIGURE 4:**
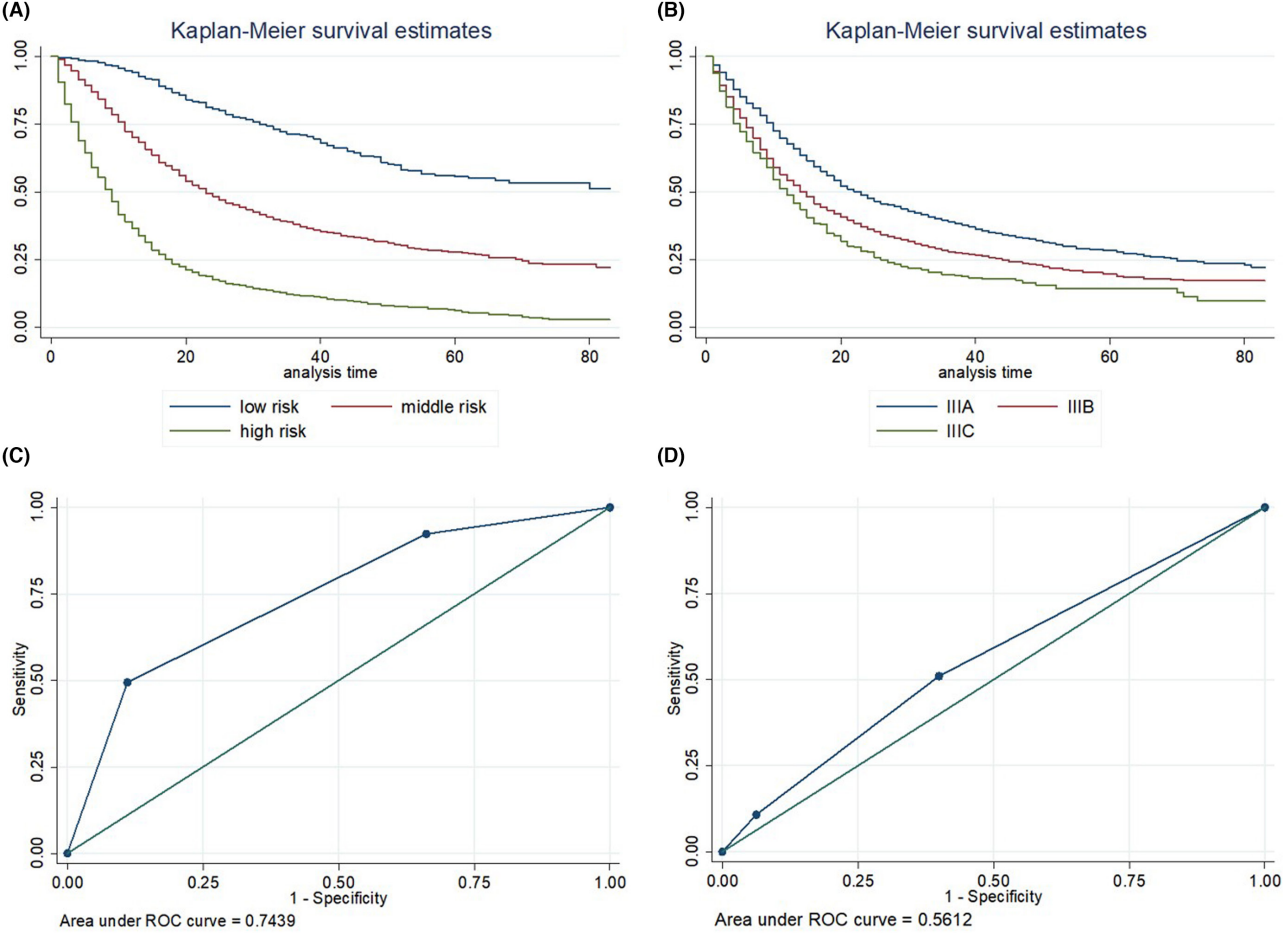
Prognostic performance of DSLM and a conventional staging system. (A) Kaplan–Meier curves for patients stratified with the new prognostic system showed a significant survival difference (log rank test, *p* < 0.05); mortality for patients in middle‐ and high‐risk groups increased 2.4‐ and 6.0‐fold, respectively, relative to patients in the low‐risk group. (B) Survival curves for patients stratified by the TNM staging system also achieved significance (*p* < 0.05), with mortality for patients with stage IIIB and IIIC disease increasing 1.3‐ and 1.7‐fold, respectively, relative to stage IIIA disease. (C) ROC curve for the new prognostic system. (D) ROC curve for the conventional staging system. DSLM, deep survival learning model; ROC, receiver operating characteristics; TNM, tumor node metastasis

Given a set of the selected features for each patient, the prognostic model was used to calculate a risk score for patients from the testing set, with a higher risk score indicating a worse prognosis. Thereafter, patients were divided into three subgroups with different prognostic statuses, namely high‐risk, middle‐risk, and low‐risk (Figure 3A). Figure 4 shows the survival curves of the test cohort for the new prognostic system (4A) and the TNM staging system (4B), where the patients showed significantly different survivals in the predicted high‐, middle‐, and low‐risk groups (log rank test, *p* < 0.05). Additionally, AUROC curves were generated to assess the model performances. The TNM staging system yielded an AUROC of 0.561 (Figure 4D), while the performance of the new prognostic system based on DSLM was significantly improved to 0.744 (Figure 4C), compared with 0.721 for RSF (Figure S4A) and 0.658 for CPH (Figure S4B). In addition, DSLM could represent follow‐up time as a series of time points, thus providing accurate survival prediction. For example, survival curves with significantly different slopes for three patients randomly selected from the testing set reflected the difference in death risks between these patients (Figure 3B).

## 4 DISCUSSION

To our knowledge, this study is the first to propose a deep learning‐based prognostic model for survival prediction in patients with stage III NSCLC. Our study was driven by the desire to develop a useful method to make accurate survival prediction and personalized treatment recommendations for patients with stage III NSCLC, which is inadequate with the currently used TNM staging system as it considers only limited variables. Yet, deep learning approaches provide a unique opportunity to cope with this challenge.

The remarkable features for stage III NSCLC are of great heterogeneity, including patients with T1‐T4 and N0‐N3, with diverse treatment strategies, pathological type, Karnofsky Performance Score, and so on. Thus, the predictive model integrated information from the distinct sources may reflect the multiple characteristics of patients and improve the predictive capability compared to TNM stage alone.^11^, ^27^ CPH is the most commonly used model for prognosis prediction of cancer patients. However, linear correlation is the essential precondition for CPH, and multidimensional parameters were commonly presented as nonlinear correlation in real world study.^28^, ^29^ Thus, deep learning, which can reveal the complex nonlinear association of parameters, provide a unique opportunity for the accurate prognosis prediction of patients with stage III NSCLC with diverse information.

As reported, Ryu SM, et al.^30^ has developed and validated a deep survival neural network for survival prediction of patients with spinal and pelvic chondrosarcoma (*n* = 1088), using clinical features extracted from the SEER database. The results indicated the significant increase of the sensitivity for survival prediction using deep learning model when compared to the conventional logistic prediction model. In another study, Song Y et al.^31^ used four different algorithms on cases from the SEER database (*n* = 3944) to train the prognosis prediction models for pancreatic neuroendocrine tumor (PNETs), among which the deep learning model showed the best predictive accuracy than logistic regression (LR), support vector machines (SVM), and random forest (RF). And the deep learning model was also found to perform better than the American Joint Committee on Cancer (AJCC) staging system (AUC = 0.87 vs. 0.76) in further comparison. It should be noted that external validation in real‐world data, which is especially important for the applicable and robustness for models, was missing in these studies.

Here, we focused on a highly heterogeneous group of patients with stage III NSCLC and trying to develop the prognosis prediction model using deep learning method. A large cohort of consecutively diagnosed stage III NSCLC cases was selected for model training and validation (*n* = 16,613) to provide more reliable results. In order to improve the unstable performance of deep learning, which is the restriction for the clinical application of these models, we further validated the robustness and general applicability of the model in an independent real‐world external dataset with the C‐index of 0.665, which suggested that it may be suitable for patients from different regions. Finally, our study found the performance of the new prognostic system was significantly superior to the TNM staging system (AUROC = 0.744 vs. 0.561), indicated that deep learning approaches are particularly suitable for processing survival information of highly heterogeneous and complex patient cohorts, while the traditional TNM staging system performs relatively poorly in such prognostic task of this patient cohorts. More importantly, our study could further provide the individualized mortality risk and survival time predictions rather than simple risk stratification, which is convenient and efficient for clinical practice.

Compared with other prognostic models, the deep learning‐based prognostic model has several advantages. First and notably, deep learning can model nonlinear risk functions and is advantageous when examining real‐life factors with complex nonlinear relationships. Second, deep learning models are able to not only directly learn feature representations from raw clinical data without making assumptions, but also fit missing data using nonlinear risk functions. Missing data pose a challenge when trying to draw clinical inferences using traditional statistical models, and may lead to serious selection bias. Third, deep learning methods are considered less vulnerable to overfitting when a large feature set is used because they are able to learn feature representation; however, the inclusion of multiple variables in conventional linear regression models may result in overfitting, which implies that a deep learning method would be more appropriate in biomedical research.

The work presented in this study lays the foundation for further research. Apart from the 15 selected factors used for stage III NSCLC survival prediction, DSLM can be expanded to integrate increasingly complex data, such as radiomics and genomics data, and predict other clinical outcomes, such as drug response and progression‐free survival (PFS). For example, immune checkpoint inhibitors (ICIs) have revolutionized cancer treatment, showing significant efficacy in NSCLC patients. The strength of the deep‐learning model may be beneficial in improving further patient selection and providing meaningful prognostic information that may be utilized in making personalized cancer treatment plans. Furthermore, although it was developed using stage III NSCLC, this deep learning‐based method can easily be generalized to other cancer types.

A limitation of this study is that the deep survival neural network has not been prospectively tested in clinical settings; however, the internal and external validation suggest the model's stability and reproducibility. Additionally, despite the black‐box nature of the end‐to‐end deep learning network, this study was successful and demonstrated that a deep learning‐based model significantly outperformed existing prognostication methods in stage III NSCLC. Inevitably, interpreting a deep learning network is challenging in clinical practice; thus, in future studies, providing clinically meaningful interpretations for deep learning models would be valuable.

## 5 CONCLUSIONS

This study demonstrated that a deep learning model may be useful for survival prediction in patients with stage III NSCLC as the model significantly outperformed existing prognostication models. This evidence suggests the utilization of this novel approach in patient stratification and personalized treatment decision‐making.

## CONFLICT OF INTEREST

The authors have no conflict of interest to declare.

## AUTHOR CONTRIBUTIONS

All authors contributed to the design of this study and to the drafting of the paper and

have seen and approved the final version.

## Supporting information


Figure S1‐S4
Table S1

## Data Availability

From the publication date, data are available upon reasonable request to the corresponding author (wanglinlinatjn@163.com).

## References

[1] Sung H , Ferlay J , Siegel RL , et al. Global cancer statistics 2020: GLOBOCAN estimates of incidence and mortality worldwide for 36 cancers in 185 countries. CA Cancer J Clin. 2021;71:209‐249. 33538338 10.3322/caac.21660

[2] Siegel RL , Miller KD , Fuchs HE , Jemal A . Cancer statistics, 2021. CA Cancer J Clin. 2021;71(1):7‐33. 33433946 10.3322/caac.21654

[3] Bradley JD , Hu C , Komaki RR , et al. Long‐term results of NRG oncology RTOG 0617: standard‐ versus high‐dose chemoradiotherapy with or without cetuximab for unresectable stage III non‐small‐cell lung cancer. J Clin Oncol. 2020;38(7):706‐714. 31841363 10.1200/JCO.19.01162 PMC7048161

[4] Walters S , Maringe C , Coleman MP , et al. Lung cancer survival and stage at diagnosis in Australia, Canada, Denmark, Norway, Sweden and the UK: a population‐based study, 2004‐2007. Thorax. 2013;68(6):551‐564. 23399908 10.1136/thoraxjnl-2012-202297

[5] Goldstraw P , Chansky K , Crowley J , et al. The IASLC lung cancer staging project: proposals for revision of the TNM stage groupings in the forthcoming (eighth) edition of the TNM classification for lung cancer. J Thorac Oncol. 2016;11(1):39‐51. 26762738 10.1016/j.jtho.2015.09.009

[6] Asamura H , Chansky K , Crowley J , et al. The international association for the study of lung cancer lung cancer staging project: proposals for the revision of the N Descriptors in the forthcoming 8th edition of the TNM classification for lung cancer. J Thorac Oncol. 2015;10(12):1675‐1684. 26709477 10.1097/JTO.0000000000000678

[7] Chaft JE , Rimner A , Weder W , Azzoli CG , Kris MG , Cascone T . Evolution of systemic therapy for stages I‐III non‐metastatic non‐small‐cell lung cancer. Nat Rev Clin Oncol. 2021;18(9):547‐557. 33911215 10.1038/s41571-021-00501-4 PMC9447511

[8] Sheikh M , Mukeriya A , Shangina O , Brennan P , Zaridze D . Postdiagnosis smoking cessation and reduced risk for lung cancer progression and mortality: a prospective cohort study. Ann Intern Med. 2021;174(9):1232‐1239. 34310171 10.7326/M21-0252

[9] Liang W , He J , Shen Y , et al. Impact of examined lymph node count on precise staging and long‐term survival of resected non‐small‐cell lung cancer: a population study of the US SEER database and a Chinese Multi‐Institutional Registry. J Clin Oncol. 2017;35(11):1162‐1170. 28029318 10.1200/JCO.2016.67.5140 PMC5455598

[10] Zuo Z , Zhang G , Song P , et al. Survival nomogram for stage IB non‐small‐cell lung cancer patients, based on the SEER database and an external validation cohort. Ann Surg Oncol. 2021;28(7):3941‐3950. 33249521 10.1245/s10434-020-09362-0

[11] Liang W , Zhang L , Jiang G , et al. Development and validation of a nomogram for predicting survival in patients with resected non‐small‐cell lung cancer. J Clin Oncol. 2015;33(8):861‐869. 25624438 10.1200/JCO.2014.56.6661

[12] Taylor JM . Random survival forests. J Thorac Oncol. 2011;6(12):1974‐1975. 22088987 10.1097/JTO.0b013e318233d835

[13] Rahman SA , Maynard N , Trudgill N , et al. Prediction of long‐term survival after gastrectomy using random survival forests. Br J Surg. 2021;108:1341‐1350. 34297818 10.1093/bjs/znab237 PMC10364915

[14] Sapir‐Pichhadze R , Kaplan B . Seeing the forest for the trees: random forest models for predicting survival in kidney transplant recipients. Transplantation. 2020;104(5):905‐906. 31403553 10.1097/TP.0000000000002923

[15] Doppalapudi S , Qiu RG , Badr Y . Lung cancer survival period prediction and understanding: deep learning approaches. Int J Med Inform. 2021;148:104371. 33461009 10.1016/j.ijmedinf.2020.104371

[16] Obermeyer Z , Emanuel EJ . Predicting the future ‐ big data, machine learning, and clinical medicine. N Engl J Med. 2016;375(13):1216‐1219. 27682033 10.1056/NEJMp1606181 PMC5070532

[17] She Y , Jin Z , Wu J , et al. Development and validation of a deep learning model for non‐small cell lung cancer survival. JAMA Netw Open. 2020;3(6):e205842. 32492161 10.1001/jamanetworkopen.2020.5842 PMC7272121

[18] Ardila D , Kiraly AP , Bharadwaj S , et al. End‐to‐end lung cancer screening with three‐dimensional deep learning on low‐dose chest computed tomography. Nat Med. 2019;25(6):954‐961. 31110349 10.1038/s41591-019-0447-x

[19] Nasrullah N , Sang J , Alam MS , Mateen M , Cai B , Hu H . Automated lung nodule detection and classification using deep learning combined with multiple strategies. Sensors (Basel) Switzerland. 2019;19(17):3722. 10.3390/s19173722 PMC6749467 31466261

[20] Shi JY , Wang X , Ding GY , et al. Exploring prognostic indicators in the pathological images of hepatocellular carcinoma based on deep learning. Gut. 2021;70(5):951‐961. 32998878 10.1136/gutjnl-2020-320930

[21] Campanella G , Hanna MG , Geneslaw L , et al. Clinical‐grade computational pathology using weakly supervised deep learning on whole slide images. Nat Med. 2019;25(8):1301‐1309. 31308507 10.1038/s41591-019-0508-1 PMC7418463

[22] Xie F , Zhang J , Wang J , et al. Multifactorial deep learning reveals pan‐cancer genomic tumor clusters with distinct immunogenomic landscape and response to immunotherapy. Clin Cancer Res. 2020;26(12):2908‐2920. 31911545 10.1158/1078-0432.CCR-19-1744 PMC7299824

[23] Robinson JT , Thorvaldsdóttir H , Wenger AM , Zehir A , Mesirov JP . Variant review with the integrative genomics viewer. Cancer Res. 2017;77(21):e31‐e34. 29092934 10.1158/0008-5472.CAN-17-0337 PMC5678989

[24] Lee C , Yoon J , Schaar MV . Dynamic‐deephit: a deep learning approach for dynamic survival analysis with competing risks based on longitudinal data. IEEE Trans Biomed Eng. 2020;67(1):122‐133. 30951460 10.1109/TBME.2019.2909027

[25] Kim DW , Lee S , Kwon S , Nam W , Cha IH , Kim HJ . Deep learning‐based survival prediction of oral cancer patients. Sci Rep. 2019;9(1):6994. 31061433 10.1038/s41598-019-43372-7 PMC6502856

[26] Fotso S. Deep Neural Networks for Survival Analysis Based on a Multi‐Task Framework. arXiv: 1801.05512v1 [stat.ML] 17 Jan 2018.

[27] Xiao HF , Zhang BH , Liao XZ , et al. Development and validation of two prognostic nomograms for predicting survival in patients with non‐small cell and small cell lung cancer. Oncotarget. 2017;8(38):64303‐64316. 28969072 10.18632/oncotarget.19791 PMC5610004

[28] Asch M , Moore T , Badia R , et al. Big data and extreme‐scale computing: pathways to convergence‐toward a shaping strategy for a future software and data ecosystem for scientific inquiry. Int J High Perform Comput Appl. 2018;32(4):435‐479.

[29] Medina‐Ortiz D , Contreras S , Quiroz C , Olivera‐Nappa Á . Development of supervised learning predictive models for highly non‐linear biological, biomedical, and general datasets. Front Mol Biosci. 2020;7:13. 32118039 10.3389/fmolb.2020.00013 PMC7031350

[30] Ryu SM , Seo SW , Lee SH . Novel prognostication of patients with spinal and pelvic chondrosarcoma using deep survival neural networks. BMC Med Inform Decis Mak. 2020;20(1):3. 31907039 10.1186/s12911-019-1008-4 PMC6945432

[31] Song Y , Gao S , Tan W , Qiu Z , Zhou H , Zhao Y . Multiple machine learnings revealed similar predictive accuracy for prognosis of pnets from the surveillance, epidemiology, and end result database. J Cancer. 2018;9(21):3971‐3978. 30410601 10.7150/jca.26649 PMC6218767

