# Supplementary material for: A novel deep learning prognostic system improves survival predictions for stage III non‐small cell lung cancer
Source: Cancer Med. 2022 May 2;11(22):4246–55. doi: 10.1002/cam4.4782 (PMC9678103; doi:10.1002/cam4.4782)
Supplement: Supplementary file 1 — Figure S1‐S4 Table S1 [file CAM4-11-4246-s001.docx]

**Supplementary Table 1. The importance ranking of all potential risk factors by RSF**

| Number | Features | Importance | Percentage importance |
| --- | --- | --- | --- |
| 0 | chemotherapy | 8.628209 | 0.109102 |
| 1 | surgery to primary site | 8.561444 | 0.108258 |
| 2 | no. of positive LN | 7.526382 | 0.095170 |
| 3 | VPI | 7.209611 | 0.091164 |
| 4 | histology | 6.413792 | 0.081101 |
| 5 | age | 6.256149 | 0.079108 |
| 6 | differentiation | 5.811485 | 0.073485 |
| 7 | T stage | 5.370007 | 0.067903 |
| 8 | radiation | 5.286260 | 0.066844 |
| 9 | sex | 4.842462 | 0.061232 |
| 10 | N stage | 4.382316 | 0.055414 |
| 11 | year of diagnosis | 3.088008 | 0.039047 |
| 12 | race | 2.737868 | 0.034620 |
| 13 | primary site | 2.317245 | 0.029301 |
| 14 | laterality | 0.652391 | 0.008249 |

Abbreviations: LN, lymph nodes; VPI, visceral pleural invasion.


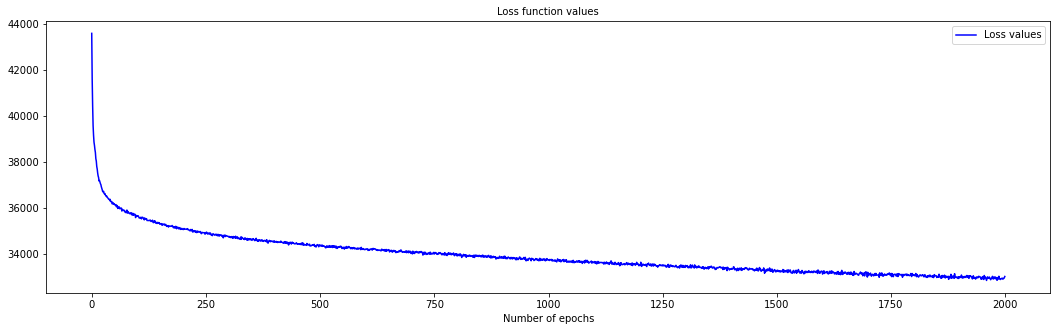


**Supplementary Figure 1. Loss function values**

The values of the loss function for DSLM decrease from 44,000 to 32,998 after 2000 epochs.


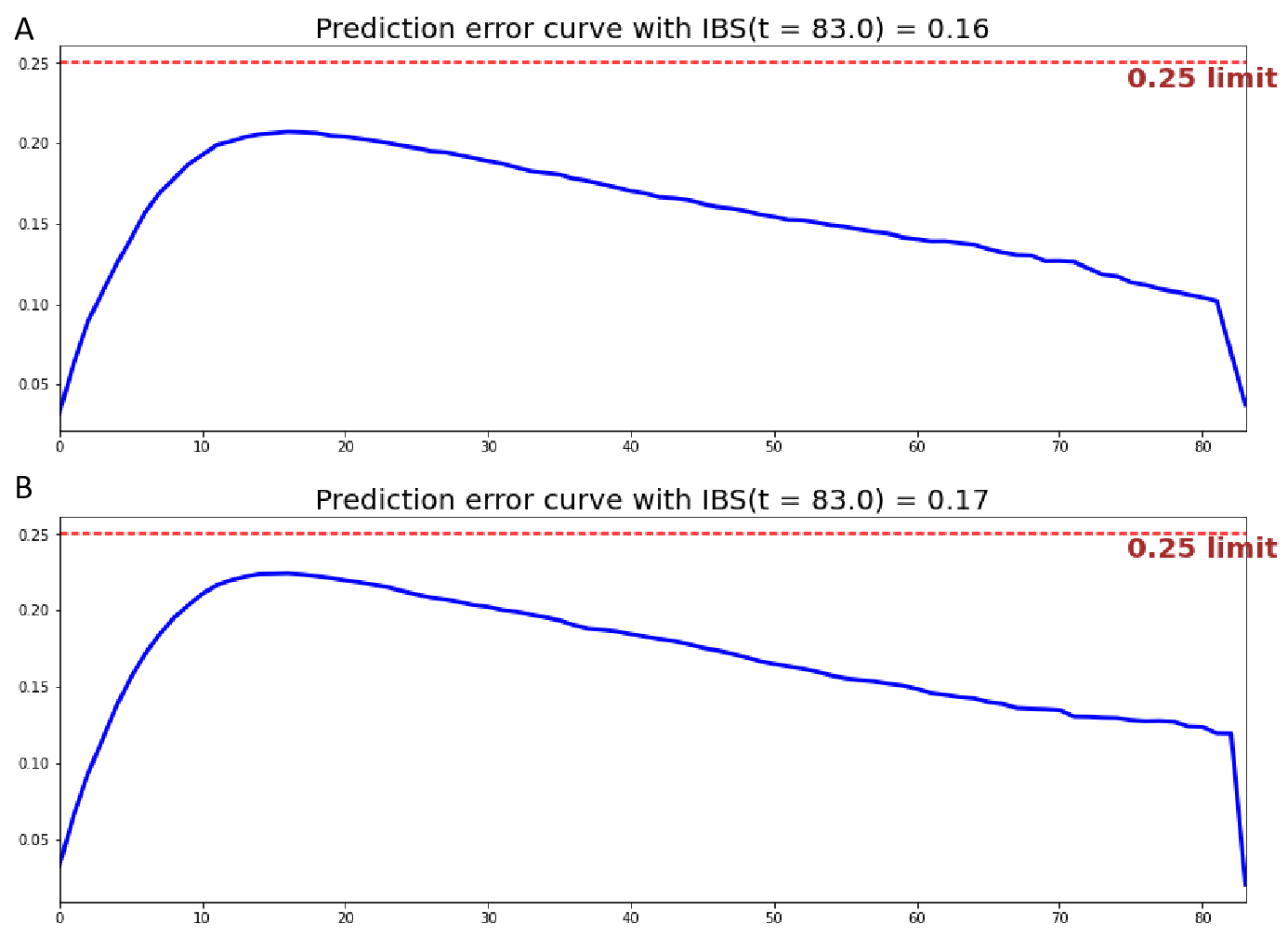


**Supplementary Figure 2. Prediction error curve with IBS for RSF and CPH**

1. RSF had an IBS of 0.16. (B) CPH had an IBS of 0.17.


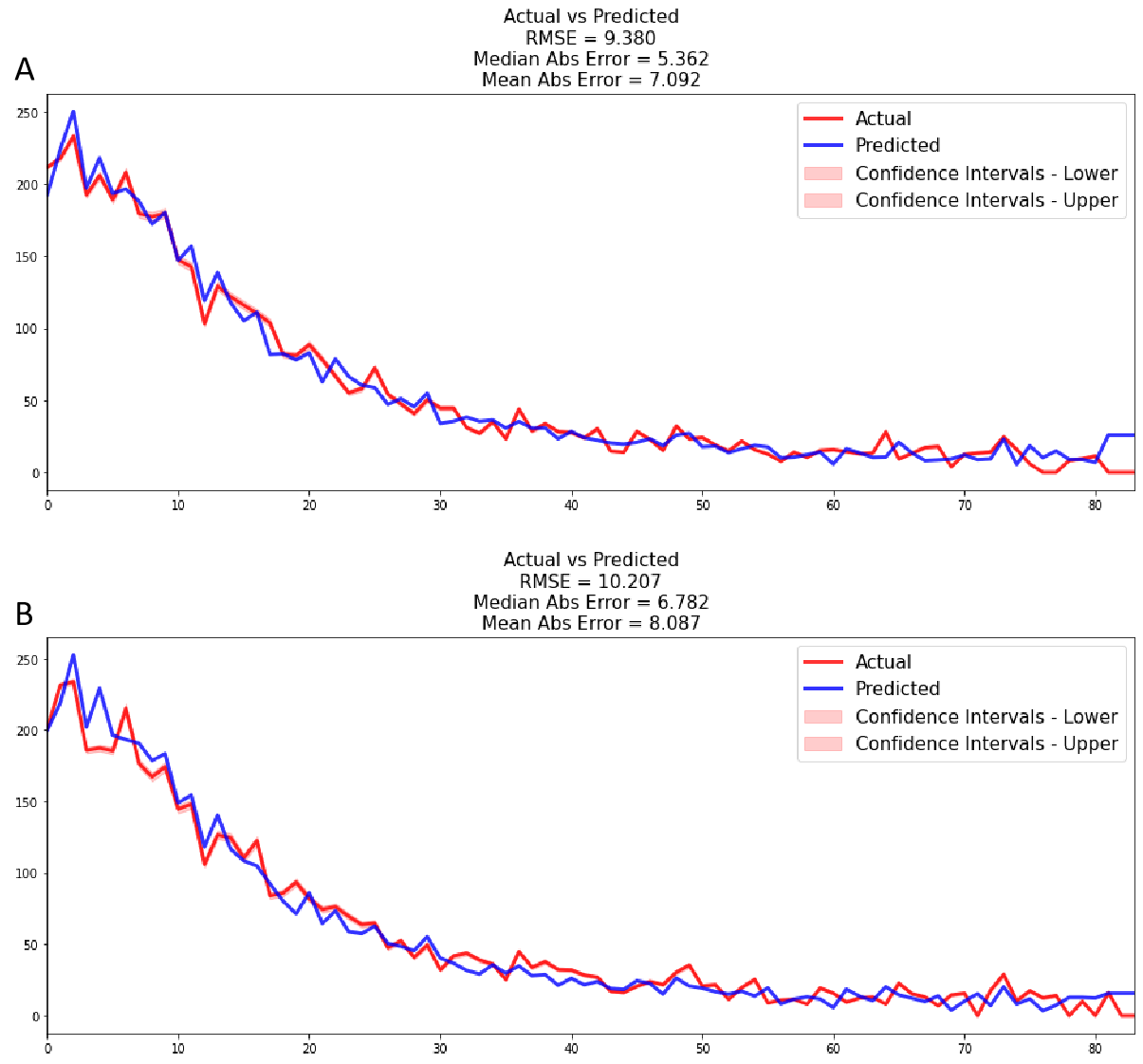


**Supplementary Figure 3. Comparation of the actual and predicted number of patient deaths over the entire follow-up**

(A) RSF yielded a median absolute error of 7.092 in the testing set. (B) CPH yielded a median absolute error of 8.087 in the testing set. The red line indicates the actual number of patient deaths, while the blue line indicated the predicted number of patient deaths.


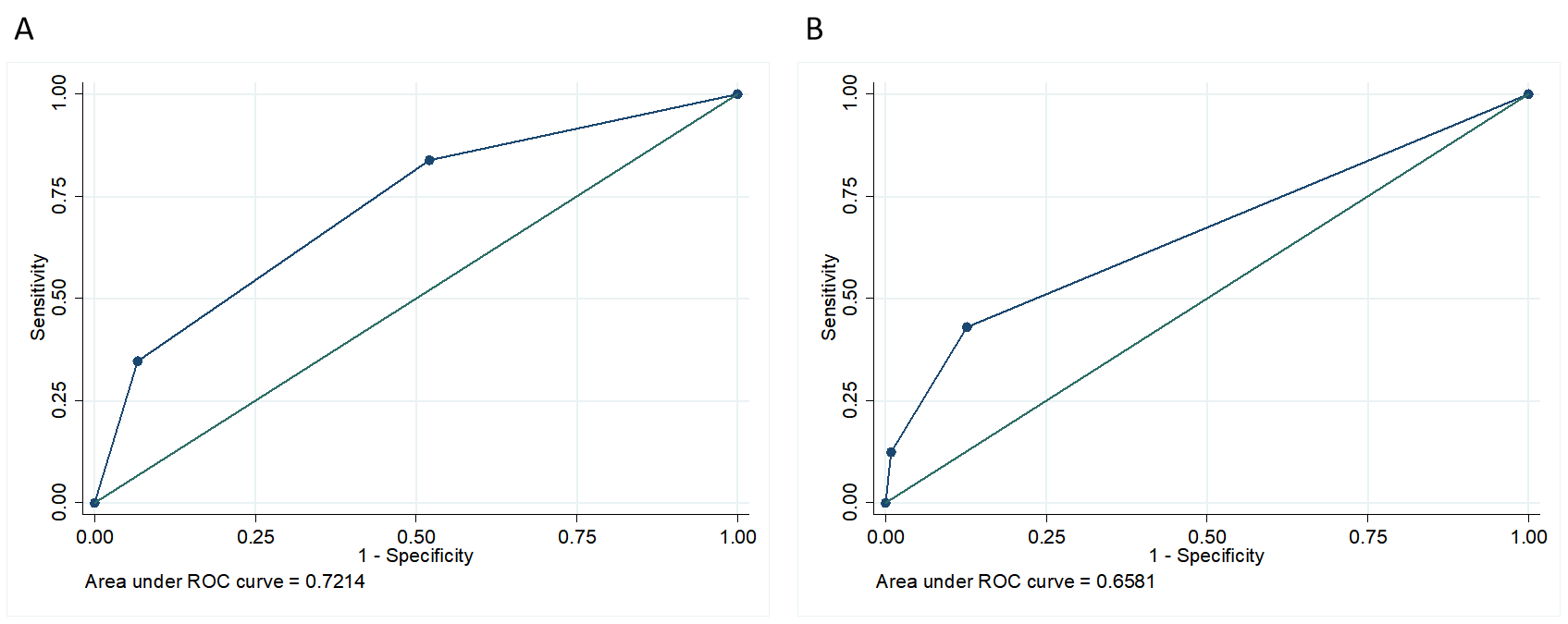


**Supplementary Figure 4. Prognostic performance of RSF and CPH**

(A) ROC curve for RSF. (B) ROC curve for CPH.
